# Intake of Flavonoids and Odds of Frailty Onset in Adults in the Framingham Offspring Cohort

**DOI:** 10.1093/geroni/igab046.3618

**Published:** 2021-12-17

**Authors:** Thuy Nga Nguyen, Courtney Millar, Douglas Kiel, Marian Hannan, Shivani Sahni

**Affiliations:** 1 University of Arizona, Tucson, Arizona, United States; 2 Hebrew SeniorLife, Roslindale, Massachusetts, United States; 3 Hebrew SeniorLife, Hebrew SeniorLife, Massachusetts, United States

## Abstract

Polyphenols (antioxidants derived from plant-foods) could play a role in inhibition of oxidative stress and frailty reduction, yet data on the polyphenol subclass of dietary flavonoids is limited. This study sought to determine the association between dietary flavonoids and frailty onset in middle-aged and older adults. This prospective cohort study included non-frail individuals from the Framingham Offspring Cohort (FOC) with total flavonoid intake (mg/day; defined as sum flavonols, flavan-3-ols, flavonones, flavones, and anthocyanins via Harvard Food Frequency Questionnaire), frailty (via Fried phenotype), and covariate information measured at baseline (1998-2001). Follow-up frailty was evaluated in 2011-2014. Logistic regression estimated odds ratio (OR) and 95% confidence intervals (95% CI) adjusting for relevant confounders. Participants (n=1,701; 55.5% female) had a mean age of 58.4 years (SD ± 8.3). Mean flavonoid intake was 309 mg/d (SD ± 266). After 12.4 years (SD ± 0.8), 224 (13.2%) individuals exhibited frailty. In age and sex adjusted models, every 50 mg/day of higher total flavonoid intake was associated with 3% reduced odds of frailty [OR (95%CI): 0.97 (0.94-1.00), p-value: 0.05). Further adjustment for smoking, energy and protein intake, and disease indicators did not appreciably change the association, and associations became non-significant (p-value=0.12). Thus, there was no association between flavonoid intake and odds of frailty onset in adults in the FOC. This could be due to participants' higher intake of flavonoids compared to average intake of ~200 mg/d in Americans.

